# Supplementary material for: Impact of the COVID-19 Lockdown on Inhaler Adherence in Patients with COPD: A South Korean Nationwide Cohort Study
Source: Healthcare (Basel). 2025 Jun 15;13(12):1431. doi: 10.3390/healthcare13121431 (PMC12193401; doi:10.3390/healthcare13121431)
Supplement: Supplementary file 1 [file healthcare-13-01431-s001.zip › Supplementary_tableS1.pdf]

Supplementary Table S1. Active principal ingredients and corresponding ATC codes of COPD inhaler medication

No.	Drug Category	Active Pharmaceutical Ingedient	ATC code
1	ICS_LABA	fluticasone propionate + salmeterol xinafoate	R03AK06
2	ICS_LABA	budesonide + formoterol fumarate dihydrate	R03AK07
3	ICS_LABA	beclomethasone dipropionate + formoterol fumarate dihydrate	R03AK08
4	ICS_LABA	fluticasone propionate + formoterol fumarate dihydrate	R03AK11
5	ICS_LABA	fluticasone furoate + vilanterol trifenatate	R03AK10
6	ICS_LABA	budesonide + salmeterol xinafoate	R03AK12
7	ICS_LABA	indacaterol acetate + mometasone furoate	R03AK14
8	LABA_LAMA	vilanterol trifenatate + umeclidinium bromide	R03AL03
9	LABA_LAMA	formoterol fumarate dihydrate + aclidinium bromide	R03AL05
10	LABA_LAMA	olodaterol hydrochloride + tiotropium bromide monohydrate	R03AL06
11	LABA_LAMA	indacaterol maleate + glycopyrronium bromide	R03AL04

Note: ICS_LABA, inhaled corticosteroids with long-acting β2-agonists; LABA_LAMA, long-acting β2-agonists with long-acting muscarinic
