# Supplementary material for: Impact of the COVID-19 Lockdown on Inhaler Adherence in Patients with COPD: A South Korean Nationwide Cohort Study
Source: Healthcare (Basel). 2025 Jun 15;13(12):1431. doi: 10.3390/healthcare13121431 (PMC12193401; doi:10.3390/healthcare13121431)
Supplement: Supplementary file 1 [file healthcare-13-01431-s001.zip › Supplementary_tableS2.pdf]

Supplementary Table S2. Underlying disease and diagnosis codes based on International Classification of Diseases, 10th revision (ICD-10) code

**Underlying diseases**	Code
**acute exacerbation in COPD**	J441
**cardiovascular disease**	I05-I09, I11, I13, I20-I25, I34-I37, I42, I43, I50, R00
**diabetes**	E10-14
**Skeletal muscle dysfunction (including osteoporosis)**	M05, M06, M15-M19, M80-M82
**mood disorder**	F30-F34, F38-F39, F40-F43, F45, F48
**lung cancer**	C33, C34
