# Supplementary material for: Impact of the COVID-19 Lockdown on Inhaler Adherence in Patients with COPD: A South Korean Nationwide Cohort Study
Source: Healthcare (Basel). 2025 Jun 15;13(12):1431. doi: 10.3390/healthcare13121431 (PMC12193401; doi:10.3390/healthcare13121431)
Supplement: Supplementary file 1 [file healthcare-13-01431-s001.zip › Supplementary_tableS3.pdf]

Supplementary Table S3. Baseline Characteristics of the Study Cohort and Changes in Inhaler Adherence Before and During the COVID-19 Lockdown

Category	Distribution		2019 PDC		2020 PDC		P value*
			Mean	SD	Mean	SD	
**TOTAL, N**	**15,971**		**56.3%**	**0.24**	**49.8%**	**0.27**	**<0.0001**
**Age, n (%)**							
40-49	427	2.67%	54.8%	0.24	50.0%	0.27	**<0.0001**
50-59	1,829	11.45%	56.8%	0.24	51.7%	0.27	
60-69	4,642	29.07%	57.3%	0.24	51.9%	0.27	
70-79	6,473	40.53%	56.5%	0.24	49.9%	0.27	
≥ 80	2,600	16.28%	53.7%	0.25	45.1%	0.29	
**Sex, n (%)**							
Female	3,316	20.76%	55.2%	0.25	48.7%	0.28	**<0.0001**
Male	12,655	79.24%	56.6%	0.24	50.2%	0.27	
**Income level, n (%)**							
Q1 (lowest)	4,923	30.82%	58.7%	0.25	52.0%	0.28	**<0.0001**
Q2	4,029	25.23%	56.0%	0.24	49.9%	0.26	
Q3	2,841	17.79%	54.9%	0.23	49.0%	0.26	
Q4 (highest)	4,178	26.16%	54.7%	0.23	47.9%	0.26	
**Insurance type, n (%)**							
NHI	12,558	78.63%	55.3%	0.24	49.0%	0.26	**<0.0001**
Medical aid	3,413	21.37%	59.9%	0.26	53.2%	0.29	
**Residential area, n (%)**							
urban	9,170	57.42%	56.0%	0.24	49.5%	0.27	**<0.0001**
rural	6,801	42.58%	56.7%	0.24	50.3%	0.27	
**Severity, n (%)**							
NO	14,236	89.14%	56.2%	0.24	49.9%	0.27	**<0.0001**
YES	1,735	10.86%	57.2%	0.24	49.8%	0.27	
**Cardiovascular disease, n (%)**							
NO	13,293	83.23%	56.9%	0.24	50.6%	0.27	**<0.0001**
YES	2,678	16.77%	53.0%	0.24	46.1%	0.26	
**Diabetes, n (%)**							
NO	13,338	83.51%	56.5%	0.24	50.1%	0.27	**<0.0001**
YES	2,633	16.49%	55.2%	0.24	48.6%	0.27	
**Musculoskeletal, n (%)**							
NO	11,479	71.87%	56.7%	0.24	50.4%	0.27	**<0.0001**
YES	4,492	28.13%	55.2%	0.25	48.5%	0.28	
**Mood disorder, n (%)**							
NO	13,946	87.32%	56.4%	0.24	50.1%	0.27	**<0.0001**

Category	Distribution		2019 PDC		2020 PDC		P value*
			Mean	SD	Mean	SD	
YES	2,025	12.68%	55.4%	0.25	48.2%	0.28	
**Lung cancer, n(%)**							
NO	15,690	98.24%	56.3%	0.24	49.9%	0.27	**<0.0001**
YES	281	1.76%	53.1%	0.24	46.4%	0.27	
**Disability, n (%)**							
NO	11,798	73.87%	55.9%	0.24	49.5%	0.27	**<0.0001**
YES	4,173	26.13%	57.3%	0.23	50.9%	0.27	
**Prescriber practice setting, n (%)**							
Hospital	8,065	50.50%	54.6%	0.23	47.8%	0.26	**<0.0001**
Clinic	7,906	49.50%	58.0%	0.25	51.9%	0.28	
**Polypharmacy, n (%)**							
<1	5,329	33.37%	58.2%	0.25	51.5%	0.28	**<0.0001**
1~2	8,094	50.68%	56.0%	0.24	49.7%	0.27	
2~3	1,947	12.19%	53.6%	0.23	47.8%	0.26	
≥3	601	3.76%	50.5%	0.24	44.4%	0.27	
**Medication treatment duration , n (%)**							
2018 year	4,659	29.17%	53.6%	0.26	46.9%	0.28	**<0.0001**
2017 year	4,430	27.74%	54.5%	0.24	48.7%	0.27	
2016 year	6,046	37.86%	58.7%	0.23	52.1%	0.26	
2015 year	836	5.23%	63.2%	0.23	56.0%	0.25	
**Inhaler type, n (%)**							
LABA_LAMA	7,146	44.74%	55.1%	0.24	48.5%	0.27	**<0.0001**
ICS_LABA	8,825	55.26%	57.2%	0.24	50.9%	0.27	

Note: PDC, proportion of days covered; LABA_LAMA, long-acting β2-agonists with long-acting muscarinic; ICS_LABA, inhaled corticosteroids with long-acting β2-agonists; *P value is under 0.05.
