# Supplementary material for: Impact of the COVID-19 Lockdown on Inhaler Adherence in Patients with COPD: A South Korean Nationwide Cohort Study
Source: Healthcare (Basel). 2025 Jun 15;13(12):1431. doi: 10.3390/healthcare13121431 (PMC12193401; doi:10.3390/healthcare13121431)
Supplement: Supplementary file 1 [file healthcare-13-01431-s001.zip › Supplementary_tableS4.pdf]

**Supplementary Table S4. Comparison of Baseline Characteristics Between High- and Low-Adherence During the COVID-19 Lockdown**

| TOTAL, N | 15,971 | | high adherence | | low adherence | | P value* |
|---|---|---|---|---|---|---|---|
| | | | 2,880 | | 13,091 | | |
| | N | % | N | % | N | % | |
| **Prior Adherence('19), n(%)** | | | | | | | **<0.0001** |
| high adherent | 3,507 | 21.96% | 2,050 | 71.2% | 1,457 | 11.1% | |
| low adherent | 12,464 | 78.04% | 830 | 28.8% | 11,634 | 88.9% | |
| **Age, n (%)** | | | | | | | **<0.0001** |
| 40-49 | 427 | 2.67% | 74 | 2.6% | 353 | 2.7% | |
| 50-59 | 1,829 | 11.45% | 351 | 12.2% | 1,478 | 11.3% | |
| 60-69 | 4,642 | 29.07% | 930 | 32.3% | 3,712 | 28.4% | |
| 70-79 | 6,473 | 40.53% | 1,124 | 39.0% | 5,349 | 40.9% | |
| ≥ 80 | 2,600 | 16.28% | 401 | 13.9% | 2,199 | 16.8% | |
| **Sex, n (%)** | | | | | | | 0.5107 |
| Female | 3,316 | 20.76% | 585 | 20.3% | 2,731 | 20.9% | |
| Male | 12,655 | 79.24% | 2,295 | 79.7% | 10,360 | 79.1% | |
| **Income level, n (%)** | | | | | | | **<0.0001** |
| Q1 (lowest) | 4,923 | 30.82% | 1,068 | 37.1% | 3,855 | 29.4% | |
| Q2 | 4,029 | 25.23% | 691 | 24.0% | 3,338 | 25.5% | |
| Q3 | 2,841 | 17.79% | 476 | 16.5% | 2,365 | 18.1% | |
| Q4 (highest) | 4,178 | 26.16% | 645 | 22.4% | 3,533 | 27.0% | |
| **Insurance type, n (%)** | | | | | | | **<0.0001** |
| NHI | 12,558 | 78.63% | 2,076 | 72.1% | 10,482 | 80.1% | |
| Medical aid | 3,413 | 21.37% | 804 | 27.9% | 2,609 | 19.9% | |
| **Residential area, n (%)** | | | | | | | **0.0165** |
| Urban | 9,170 | 57.42% | 1,596 | 55.4% | 7,574 | 57.9% | |
| Rural | 6,801 | 42.58% | 1,284 | 44.6% | 5,517 | 42.1% | |
| **Severity, n (%)** | | | | | | | 0.2373 |
| NO | 14,236 | 89.14% | 2,585 | 89.8% | 11,651 | 89.0% | |
| YES | 1,735 | 10.86% | 295 | 10.2% | 1,440 | 11.0% | |
| **Disability, n (%)** | | | | | | | 0.4971 |
| NO | 11,798 | 73.87% | 2,113 | 73.4% | 9,685 | 74.0% | |
| YES | 4,173 | 26.13% | 767 | 26.6% | 3,406 | 26.0% | |
| **Cardiovascular disease , n (%)** | | | | | | | **<0.0001** |
| NO | 13,293 | 83.23% | 2,510 | 87.2% | 10,783 | 82.4% | |
| YES | 2,678 | 16.77% | 370 | 12.8% | 2,308 | 17.6% | |
| **Diabetes, n (%)** | | | | | | | 0.1371 |
| NO | 13,338 | 83.51% | 2,432 | 84.4% | 10,906 | 83.3% | |
| YES | 2,633 | 16.49% | 448 | 15.6% | 2,185 | 16.7% | |

| TOTAL, N | 15,971 | | high adherence | | low adherence | | P value* |
| --- | --- | --- | --- | --- | --- | --- | --- |
| | | | 2,880 | | 13,091 | | |
| **Musculoskeletal, n (%)** | | | | | | | **0.0439** |
| NO | 11,479 | 71.87% | 2,114 | 73.4% | 9,365 | 71.5% | |
| YES | 4,492 | 28.13% | 766 | 26.6% | 3,726 | 28.5% | |
| **Mood disorder, n (%)** | | | | | | | 0.1704 |
| NO | 13,946 | 87.32% | 2,537 | 88.1% | 11,409 | 87.2% | |
| YES | 2,025 | 12.68% | 343 | 11.9% | 1,682 | 12.8% | |
| **Lung cancer, n (%)** | | | | | | | 0.1300 |
| NO | 15,690 | 98.24% | 2,839 | 98.6% | 12,851 | 98.2% | |
| YES | 281 | 1.76% | 41 | 1.4% | 240 | 1.8% | |
| **Prescriber practice setting , n (%)** | | | | | | | **<0.0001** |
| Hospital | 8,065 | 50.50% | 1,186 | 41.2% | 6,879 | 52.5% | |
| Clinic | 7,906 | 49.50% | 1,694 | 58.8% | 6,212 | 47.5% | |
| **Polypharmacy, n (%)** | | | | | | | **<0.0001** |
| <1 | 5,329 | 33.37% | 1,110 | 38.5% | 4,219 | 32.2% | |
| 1~2 | 8,094 | 50.68% | 1,415 | 49.1% | 6,679 | 51.0% | |
| 2~3 | 1,947 | 12.19% | 283 | 9.8% | 1,664 | 12.7% | |
| ≥3 | 601 | 3.76% | 72 | 2.5% | 529 | 4.0% | |
| **Medication treatment duration, n (%)** | | | | | | | **<0.0001** |
| 2018 year | 4,659 | 29.17% | 766 | 26.6% | 3,893 | 29.7% | |
| 2017 year | 4,430 | 27.74% | 753 | 26.1% | 3,677 | 28.1% | |
| 2016 year | 6,046 | 37.86% | 1,171 | 40.7% | 4,875 | 37.2% | |
| 2015 year | 836 | 5.23% | 190 | 6.6% | 646 | 4.9% | |
| **Inhaler type, n (%)** | | | | | | | **<0.0001** |
| LABA_LAMA | 7,146 | 44.74% | 1,193 | 41.4% | 5,953 | 45.5% | |
| ICS_LABA | 8,825 | 55.26% | 1,687 | 58.6% | 7,138 | 54.5% | |

Note: LABA_LAMA, long-acting β2-agonists with long-acting muscarinic; ICS_LABA, inhaled corticosteroids with long-acting β2-agonists; **P* value is under 0.05.
